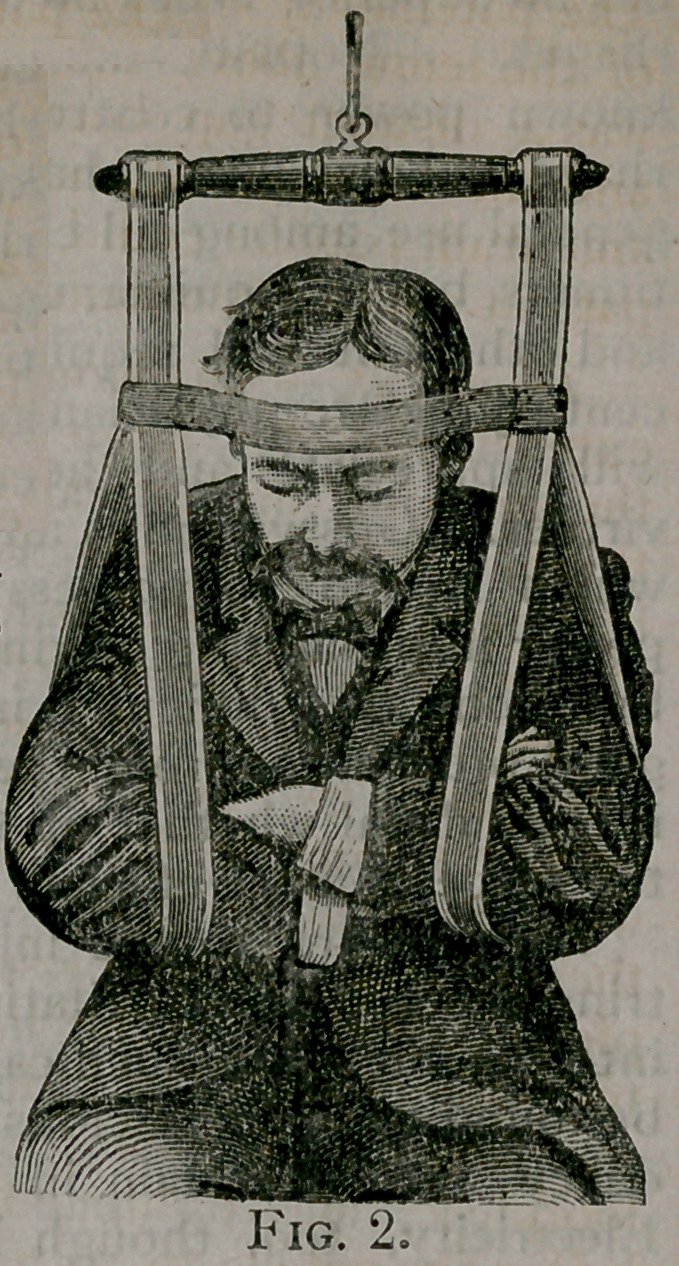# A Respiratory Brace

**Published:** 1877-10-20

**Authors:** 


### A RESPIRATORY BRACE.

Dr. Geo. F. French, of Portland, Me., has devised (*Boston Medical and Surgical Journal*) a very useful appliance for the relief of orthopnœa. It consists, as will be seen from the accompanying cuts (Figs. 1 and 2), of a cross-bar, from the extremities of which hang two loops of strong elastic webbing for the support of the shoulders. The broad band encircling the head is steadied by guys stretching across on both sides to the upright elastic supports. The apparatus is suspended by a pulley or ring from the ceiling. Fig 1 represents a patient resting with the brace applied under the shoulders. Whenever, from the helplessness or weight of the patient, or from the tedious duration of the case, the circulation in the arm is impeded, the support should be afforded by the elbows, as in Fig. 2, in which the entire pressure comes upon the outside of the forearm. Usually, however, the degree of pressure under the arms requisite to sustain a person who is sitting is insufficient to interfere with the circulation. The utilility of the apparatus can hardly be questioned. The thorax being slung as it were by the arm-pits, and the head properly steadied, muscular fatigue is prevented, the voluntary and involuntary respiratory muscles have the best possible chance to act, and the patient is supported in an easy sitting position for sleep. Aside from this, the apparatus can be adapted to every case in which it is necessary to afford an effectual and comfortable sitting position and relieve the upper portion of the body from the weight of the shoulders and head.—*N. Y. Medical Record.*

## Figures and Tables

**Fig. 1. f1:**
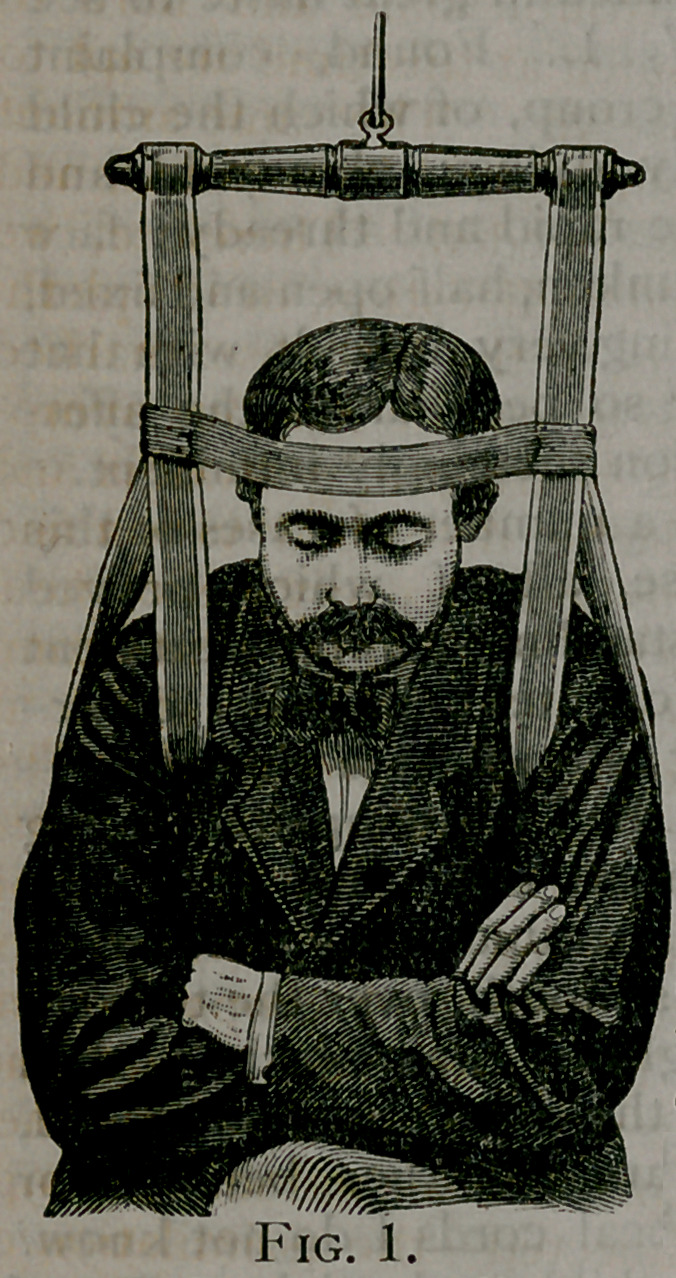


**Fig. 2. f2:**